# Obstructive jaundice, early satiety and xerostomia in an 82-year-old woman

**DOI:** 10.1093/jscr/rjac264

**Published:** 2022-07-08

**Authors:** Kunal Bhanot, Muhesh Taheem, Robert Perry, Christopher Wong, Charlotte Muehlschlegel, Kuldeep Cheent

**Affiliations:** Department of Gastroenterology, Frimley Park Hospital, Portsmouth Road, Frimley, Camberley, UK; Department of Surgery, North Middlesex University Hospital, Sterling Way, London, UK; Department of Gastroenterology, Frimley Park Hospital, Portsmouth Road, Frimley, Camberley, UK; Department of Gastroenterology, Frimley Park Hospital, Portsmouth Road, Frimley, Camberley, UK; Department of Surgery, North Middlesex University Hospital, Sterling Way, London, UK; Department of Gastroenterology, Frimley Park Hospital, Portsmouth Road, Frimley, Camberley, UK

## Abstract

Amyloid light-chain (AL) amyloidosis is a multisystem disease with obstructive jaundice and gastrointestinal (GI) involvement being uncommon initial presentations. Patients with AL amyloidosis seldom have jaundice and advanced GI tract involvement as their presenting symptoms. This case report describes an 82-year-old lady who presented with a 6-month history of early satiety, weight loss, xerostomia and progressive jaundice. Imaging did not suggest a biliary cause but demonstrated hepatomegaly and ascites. Oesophagogastroduodenoscopy revealed a duodenal stricture. Duodenal and liver biopsies were consistent with amyloid deposition. Multiple myeloma was confirmed to be the underlying cause. Significant cholestatic liver dysfunction and a duodenal stricture have not been previously described as simultaneous manifestations of amyloidosis. This case also highlights the difficulty in treating multiple myeloma as the cause of AL amyloidosis in the context of liver dysfunction, given that many chemotherapy agents undergo hepatic metabolism.

## INTRODUCTION

An 82-year-old woman was referred for an urgent oesophagogastroduodenoscopy (OGD) on the basis of early satiety and weight loss. She had a background of type 2 diabetes mellitus but was otherwise fit and well. Her OGD revealed a normal oesophagus, erosive gastritis and an ulcerated duodenal stricture from which biopsies were taken. Over the next few weeks she developed rapidly worsening jaundice with derangement of her liver function tests and was subsequently admitted to hospital for further assessment. At this stage, she also reported some difficulty in eating due to a dry mouth and burning sensation, particularly affecting her tongue.

## CASE REPORT

On examination she was visibly cachectic and jaundiced. Oral cavity examination was normal and there were no obvious skin lesions. Her abdomen was soft and non-tender with smooth hepatomegaly on palpation. There was no evidence of ascites or encephalopathy. She had an ejection systolic murmur radiating to the carotid arteries and no peripheral oedema. Respiratory system examination was normal.

Her initial laboratory investigations showed a raised total bilirubin (258 μmol/L), alkaline phosphatase (814 U/L), gamma-glutamyltransferase (725 U/L), alanine aminotransferase (163 U/L), aspartate aminotransferase (64 U/L), prolonged prothrombin time (20.1 s) and raised N-terminal prohormone brain natriuretic peptide (7178 ng/L). Abdominal ultrasound scan revealed an enlarged liver with subtle surface irregularity, mild to moderate ascites and normal portal venous flow. There was no biliary dilatation or other abnormalities noted. Computed tomography of the chest, abdomen and pelvis was unremarkable. Magnetic cholangiopancreatography did not show any obvious large bile duct obstruction or cholangiopathy. Non-invasive liver screen was unremarkable and tumour markers were within normal range.

**Figure 1:**
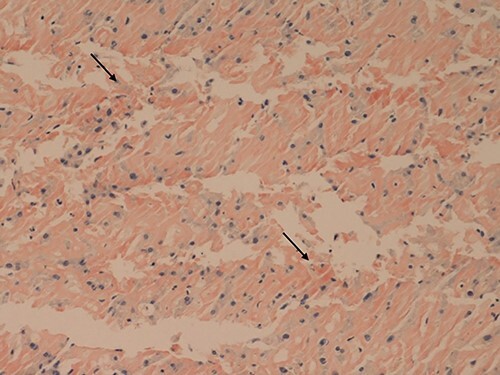
Liver core biopsies (Congo red, x20): the amorphous material in the sinusoids demonstrates salmon pink staining under standard light microscopy in keeping with amyloid deposition.

**Figure 2:**
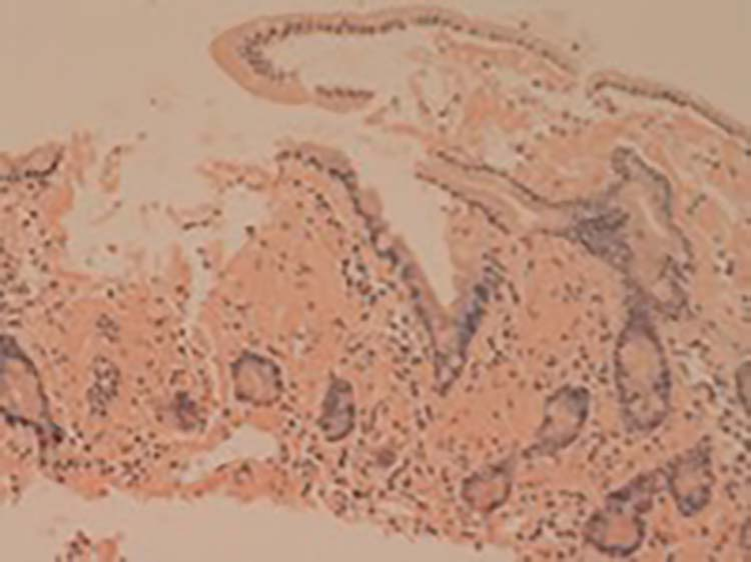
Small bowel mucosa (Congo red, x20): the substance is positive with Congo red stain in keeping with amyloid (salmon pink staining under standard light microscopy).

Her liver biochemistry continued to deteriorate so a transjugular liver biopsy was performed. Histology from both liver and duodenal biopsies was consistent with amyloidosis on Congo red staining (Figs 1 and 2, respectively). A subsequent bone marrow biopsy, organized by the haematologists, revealed a low level infiltrate of plasma cells consistent with multiple myeloma.

## DISCUSSION

This lady had multiple myeloma causing advanced amyloid light chain (AL) amyloidosis with large liver, splenic and bone load. This was diagnosed after bone marrow biopsy revealed 35–40% plasma cells in conjunction with an IgG kappa paraprotein of 13 g/L and kappa light chain to lambda light chain ratio of 8.01. In addition, she displayed clinical features of oral amyloidosis manifesting as xerostomia and tongue burning. Her significantly raised N-terminal prohormone brain natriuretic peptide was also suggestive of cardiac involvement.

Amyloidosis is a disorder characterized by abnormal deposition of insoluble amyloid fibrils in tissues, thus disrupting their normal function. Many forms of amyloidosis have been documented, however AL and amyloid A (AA) variants remain the most common entities. Deposition can be local or systemic and can produce an array of clinical syndromes [1].

The chronology of organ system involvement in systemic amyloidosis is impossible to demarcate, but oral amyloidosis was likely an early feature in this case. Oral amyloidosis is an uncommon condition, where the tongue is most commonly involved followed by the buccal mucosa, soft palate, hard palate and gingiva [2]. Salivary gland, pharyngeal, laryngeal and orbital sinus amyloidosis have also been previously described. Interestingly, there were no obvious tongue lesions on gross examination, whereas previous cases describe ‘rubbery’ nodules affecting the lateral borders of the tongue [2].

Coexisting upper gastrointestinal tract (UGI) amyloid deposition alongside oral involvement made this an unusual case. Endoscopic findings initially revealed a narrowed lumen around the second part of the duodenum. This phenomenon was consistent with the thickened mucosal folds documented by Iida *et al*. [3] in their review of endoscopic findings in patients with established amyloidosis. AA amyloidosis, in contrast, can display mucosal friability and a fine granular appearance; such patients are more susceptible to UGI bleeding. It is also important to note that polypoid protrusions are evident in AL amyloidosis, which was not the case here, perhaps indicating early stage mucosal involvement only.

Finally, this lady also had severe hepatic amyloidosis culminating in cholestatic liver dysfunction. Liver injury in amyloidosis is characterized by amorphous amyloid material flooding sinusoids causing stasis of bile. Interestingly, AL amyloidosis yields far greater hepatic deposition than the AA form but there is no clear correlation between extent of deposition and biochemical abnormality [1]. AL amyloidosis is the most common form of systemic amyloidosis but it only occurs in 5–15% of patients with multiple myeloma [4]. Moreover, liver involvement is less common in multiple myeloma compared with other haematological malignancies. Patients often present with jaundice, hepatomegaly and ascites, as was seen here.

Deranged liver enzymes and elevated total serum bilirubin in the context of primary amyloidosis carry a poor prognosis [1]. One of the major challenges with significant hepatic involvement is choosing a chemotherapy agent that does not exacerbate hepatic impairment. However, chemotherapy agents have been used in the past with success, albeit on a case by case basis. Sadhegi *et al*. [5] reported a 52-year-old man with subacute liver failure secondary to multiple myeloma who responded to a 2-month course of bortezomib, thalidomide and dexamethasone. In our case, a similar regimen of bortezomib and dexamethasone was initially started to slow disease progression. Cyclophosphamide was not utilized as the liver would have been unable to metabolize the active moiety. She was however readmitted to hospital due to worsening malaise, confusion and jaundice. A palliative approach was thus taken and she died within a week of readmission.

## CONCLUSION

This case highlights a rare presentation of amyloidosis with advanced cholestatic liver dysfunction and failure, gastrointestinal tract involvement and likely oral involvement; the first of its kind. Liver involvement has been previously described, but is usually clinically insignificant with studies reporting incidental deposits in a large proportion of patients who reported no symptoms. The presence of severe intrahepatic cholestatic jaundice is a poor prognostic sign, as is the development of ascites [4]. Moreover, treating multiple myeloma in the context of hepatic dysfunction can be challenging. Recognition of symptoms and early diagnosis is therefore essential to prevent progressive organ involvement. In practice, this remains difficult, given the non-specific nature of systemic amyloidosis and vast collection of initial clinical presentations.
